# The different faces of mass action in virus assembly

**DOI:** 10.1007/s10867-018-9487-6

**Published:** 2018-04-03

**Authors:** Bart van der Holst, Willem K. Kegel, Roya Zandi, Paul van der Schoot

**Affiliations:** 1 Department of Applied Physics, Eindhoven University of Technology, Eindhoven, The Netherlands; 2 Department of Chemistry, Utrecht University, Utrecht, The Netherlands; 3 Department of Physics and Astronomy, University of California Riverside, Riverside, USA; 4 Institute for Theoretical Physics, Utrecht University, Utrecht, The Netherlands

**Keywords:** Virus coat proteins, Oligo and polynucleotides, Co-assembly, Law of mass action, Overcharging, Competition, Parasitic binding to host proteins

## Abstract

The spontaneous encapsulation of genomic and non-genomic polyanions by coat proteins of simple icosahedral viruses is driven, in the first instance, by electrostatic interactions with polycationic RNA binding domains on these proteins. The efficiency with which the polyanions can be encapsulated *in vitro*, and presumably also *in vivo*, must in addition be governed by the loss of translational and mixing entropy associated with co-assembly, at least if this co-assembly constitutes a reversible process. These forms of entropy counteract the impact of attractive interactions between the constituents and hence they counteract complexation. By invoking mass action-type arguments and a simple model describing electrostatic interactions, we show how these forms of entropy might settle the competition between negatively charged polymers of different molecular weights for co-assembly with the coat proteins. In direct competition, mass action turns out to strongly work against the encapsulation of RNAs that are significantly shorter, which is typically the case for non-viral (host) RNAs. We also find that coat proteins favor forming virus particles over nonspecific binding to other proteins in the cytosol even if these are present in vast excess. Our results rationalize a number of recent *in vitro* co-assembly experiments showing that short polyanions are less effective at attracting virus coat proteins to form virus-like particles than long ones do, even if both are present at equal weight concentrations in the assembly mixture.

## Introduction

A fair number of cylindrical and spherical single stranded RNA viruses have been reconstituted *in vitro* [1, 2]. This suggests that the underlying processes leading to virus assembly are physical in nature and not necessarily tied to the biochemical machinery of susceptible host cells [3]. For spherical viruses, the principal driving force seems to be electrostatic interactions between the negatively charged RNA and positively charged disordered RNA-binding domains on the coat proteins [4–7], also known as arginine rich motifs or ARMs [8–10]. A secondary but nonetheless still crucial driving force, guiding the proteins to build an icosahedral shell called the capsid around the polynucleotide on which they condense, are lateral interactions between the proteins [4, 11–13]. These seem to include hydrophobic interactions, hydrogen bonds and complexes involving ionic species [14–16]. In addition, specific interactions between coat proteins and so-called packaging signals on the genome may facilitate encapsulation by inducing conformational switching and prevent kinetic trapping into aberrant particles [17–19].

Over the past two decades, a large number of experimental, theoretical and simulation studies have appeared that aim to further our understanding of the basic principles that underpin the assembly of simple viruses [9, 11, 12, 20–28]. From these, it is has become evident that the idea that while encapsulation must indeed be driven by electrostatics because the coat proteins of a variety of viruses readily encapsulate heterologous RNAs [29], synthetic polyanions [30], and negatively charged nanoparticles [21], the underlying physics must be much more complex and rich [31]. For instance, there is the important issue of the conformational statistics of a polymeric cargo that needs to be condensed in a relatively small volume of space [4, 7]. Of importance is also the secondary structure of viral RNAs that has been suggested to strongly favor encapsulation by the virus coat proteins making them relatively compact [20, 32, 33]. On the other hand, it has in addition become clear that even though the coat proteins in a virus shell or capsid may have a preferred curvature, they also exhibit some degree of flexibility when it comes to size selection and accommodating their cargo [21, 30, 34–36].

All in all, a complex supramolecular free energy landscape emerges needed to describe what the optimal molecular weight is for a particular type of polyanionic cargo and what the associated optimal capsid size (and shape) must be [12, 31, 35]. Provided the co-assembly is reversible and not dominated by kinetics [13, 37–39], an assumption supported by the work of Zlotnick who showed that the assembly of many spherical viruses follows a reversible path, see Ref. [40] and references cited therein, it is mass action that in the end determines how this free energy landscape expresses itself in the optimal final product [11]. This means that the concentrations of all constituents, absolutely and relatively, have an impact on what the precise outcome of an assembly experiment is [35], a circumstance that perhaps is not yet widely appreciated.

Indeed, concentration and stoichiometry seems to play an often ignored role in size selection, and hence in polymorphism [35], and should also be important in the competition of various species of polyanion for encapsulation by coat proteins [41]. The latter could be relevant in the context of *in vivo* encapsulation, because the cytosol is awash with mRNAs that arguably compete with viral RNAs for complexation [41]. In fact, the cytosol is also awash with other proteins that could non-specifically bind to coat proteins, if only because a very large fraction of proteins present in the cytosol carry a net negative charge and, in principle, compete with virus assembly. Of course, if virus assembly is compartimentalized in, e.g., virus factories, then parasitic binding of coat proteins to cellular RNAs and proteins must be less important [42].

In this paper, we illustrate the role that mass action may have in different aspects of the assembly of viruses and virus-like particles, where virus-like particles consist of virus coat proteins and non-native polynucleotides or synthetic polyanionic cargo. We first illustrate, in Section 2, how replacing a single polyanionic cargo by multiple copies of equal total length decreases the free energy gain and thus destabilises co-assembly. This happens to be so, even if we keep the overall binding free energy of a virus-like particle fixed, as well as the mass concentrations of the protein and the cargo in the solution. More specifically, if we keep the total mass of the polynucleotides constant in the capsid but vary their lengths, we find that the connectivity of the polyanionic cargo strongly contributes to the stability of the supramolecular complex. Our findings rationalize recent experimental findings that we will discuss in some detail.

Next, in Section 3, we apply the classic Voorn-Overbeek theory for the complex coacervation of oppositely charged polymers to the binding of polyanions to the ARM region lining the inner wall of a capsid. Focusing attention on relatively short polyanionic cargo molecules, we find that the overall binding free energy of a complex need not be equal if the polyanions are of different molecular weight [43, 44]. In fact, the overall binding free energy depends not only on the molecular weight but also the background concentration of polyanions in the solution: it becomes more negative with increasing concentration. Hence, in a direct competition, what species finds itself preferentially encapsulated by coat proteins depends not only on the number of charges on it but also this background concentration. In this respect, the co-assembly of coat proteins and nucleic acids is not all that different from adsorption of a polymer onto a surface.

Finally, we investigate in Section 4 competing binding of coat proteins to non-viral proteins, and show that the concentration of the non-viral proteins needs to be exceedingly large for virus assembly to be strongly affected. This is caused by differences in how free energies and concentrations enter binding isotherms: the former in the form of a Boltzmann weight, so exponentially, the latter only algebraically. As a consequence, differences in binding free energy of only a few times the thermal energy already require large concentration differences to compensate for. Arguably, binding free energies of single coat proteins in complete virus particles are very much larger than that with random host cell proteins also present in the cytosol, explaining why parasitic binding cannot strongly affect virus assembly.

We end the paper with a discussion and conclusions in Section 5, where we also outline how a more complete theory describing the co-assembly of polyanions and virus coat proteins may be set up.

## Cargo length and encapsulation efficiency

Recent experiments by Cornelissen et al. have shown that if polyanions encapsulated by the virus coat proteins of cowpea chlorotic mottle virus (CCMV) are depolymerised by UV irradiation, the virus-like particles will collapse [45], which could suggest that connectivity of the encapsulated polymer impacts upon the stability of the virus shell for a given total amount of encapsulated charge. Indeed, recent experiments by a number of groups indicate that polyanions are less efficiently encapsulated if their molecular weight is very much smaller than that of the native genome [20, 46, 47]. On the face of it, this may seem not entirely surprising given that the loss of translational and mixing entropy, if measured per unit weight of assembled material, increases with increasing number of components involved. On the other hand, this does presume that the thermodynamic driving force for assembly remains equal, which is not necessarily the case. Indeed, equal mass concentrations of polyanions with different molecular weights produces a different thermodynamic driving force for these polyanions, because mass action is governed by the number concentration, not the mass concentration.

Another issue that has not yet received a lot of attention in the theoretical virus physics literature is how the interaction between the polyanions and the RNA binding domains or ARMs is affected by the molecular weight of the former. Indeed, density functional theoretic calculations are usually done at the level of the ground-state approximation, which presumes all segments to be statistically equivalent [23, 48–52]. This implies that end- or finite-size effects are ignored. Arguably this is a good approximation if the RNA binding domain is very much smaller than the unperturbed radius of gyration of the polymer in free solution [4]. In practice, this is the case when a single or a few polyanions are encapsulated, because the number of negative charges encapsulated is of the order of the total number of positive charges on the RNA-binding domains in a complete virus or virus-like particle [32]. This number typically exceeds about one thousand even for the smallest viruses [53]. The ground-state approximation should be treated with some skepticism, however, if the polyanions are not very much larger than the RNA-binding domains that involve fewer than a hundred or so amino acids, but typically no more than a few dozen [6, 29].

Let us, for simplicity, ignore this complication for the moment, and pretend that the free energy of encapsulating the optimal number of polyanionic charges is a weak function of their molecular weight. We return to this issue in the next section. We assume that an optimal virus-like particle co-assembles *q* coat proteins and *p* polyanions. Because for icosahedral viruses *q* = *T* × 60 with *T* = 1,3,4,7,… the triangulation number, *q* should be considered a large number. The fact that this is a large number makes the co-assembly a highly co-operative process [32]. The quantities *p* and *q* are related to what is known as the degree of overcharging [8]. If *N*_pc_ denotes the number of positive charges on the ARM of each coat protein, and *N*_pa_ the number of negative charges on a single encapsidated chain, then *Q* ≡ *pN*_pa_/*qN*_pc_ is defined as the degree of overcharging. It has been argued that for viruses, on average, *Q* ≃ 1.6 albeit that the spread around this value is substantial [5, 6, 54]. Experiments on synthetic polyions and nanoparticles find *Q* is between 0.6 and 9, depending on the molecular weight, topology of the polyanion and so on [30, 55, 56]. Recently, we argued that the value of *Q* should depend on the level of (annealed) branching, the linear charge density and the quality of the solvent of the polyanion [8, 32, 49, 50, 52]. In the next section, we make it plausible that in addition *Q* may well depend on the concentration of the polyanion too.

We define *c*_cp_ as the (fixed) total mole fraction coat proteins and *c*_pa_ the (fixed) total polymer mole fraction present in the solution. Let *q* coat proteins encapsulate *p* polyanions, that is, for a given length of polymer, the optimal number of encapsulated negatively charged polymers or polyanions is *p*. Clearly, the optimal number of polyanions, *p*, depends on the total number of charges on each chain, which is proportional to its molecular weight. According to the law of mass action, obtained by equating the chemical potentials of the components in the assembly and those in free solution, the equilibrium value of the mole fraction of virus-like particles *ρ*_vlp_ is a function of the equilibrium value of the mole fraction of coat proteins free in solution, *ρ*_cp_, and the equilibrium value of the mole fraction of polyanions free in solution, *ρ*_pa_, according to [57]

1 $$ \rho_{\text{vlp}} = \rho_{\text{pa}}^{p} \rho_{\text{cp}}^{q} \exp \left( -\beta {\Delta} G \right), $$

at least in dilute solution, where Δ*G* = Δ*G*(*p*,*q*) is overall binding free energy of *p* polyanions and *q* coat proteins into a single virus-like particle, and *β* the usual reciprocal thermal energy. Conservation of mass demands that *c*_cp_ = *ρ*_cp_ + *qρ*_vlp_ and *c*_pa_ = *ρ*_pa_ + *pc*_vlp_. This allows us to rewrite (1) in terms of the experimental control variables *c*_cp_ and *c*_pa_, and the quantity Δ*G*(*p*,*q*). Below we link Δ*G*(*p*,*q*) to a critical assembly concentration, which makes it an observable. We stress that the concentrations indicated by the mole fractions *ρ* are actually expectation values, so vary with the solution conditions.

Experiments are often performed under conditions of constant stoichiometry, so at constant *c*_pa_/*c*_cp_, and increasing the concentration of both constituents, or at fixed concentrations and changing the solution conditions. Alternatively, *c*_pa_/*c*_cp_ can be varied and either *c*_pa_ or *c*_cp_ kept constant as well as the solution conditions. These two modes of operation lead, not surprisingly, to very different titration behaviour [34]. We shall restrict ourselves to the case of constant stoichiometry. Recent experiments are perfomed, for instance, under the condition that *c*_pa_/*c*_cp_ is fixed to achieve overall charge neutrality [47]. This means that *c*_pa_/*c*_cp_ = *N*_pc_/*N*_pa_ = *p*/*Qq*. Knowing that *Q* is between one and two for RNAs we for simplicity set it equal to one. Our results would not change qualitatively if we were to depart from this value, except if we were to take the limits *Q* ≪ 1 or *Q* ≫ 1. For the purposes of our discussion, these limits are not of any importance.

If we set *c*_pa_/*c*_cp_ = *p*/*q* and the fraction of coat proteins in capsid form *η* ≡ *qρ*_vlp_/*c*_cp_, (1) becomes,

2 $$ \eta = q c_{\text{cp}}^{p+q-1}\left( \frac{p}{q} \right)^{p} \left( 1-\eta\right)^{p+q} \exp \left( -\beta {\Delta} G \right)  $$

Making use of the limit *q* ≫ 1, where *q*^1/(*p* + *q*)^ → 1 and *η*^1/(*p* + *q*)^ → 1 unless *η* is exceedingly small, we deduce from (2) that for *c* ≥ *c*_∗_,

3 $$ \eta = 1- \left( \frac{c_{*}}{c_{\text{cp}}} \right), $$

holds, with

4 $$ c_{*} = \left( \frac{p}{q} \right)^{-\frac{p}{p+q}}\exp \left( \frac{\beta {\Delta} G}{p+q} \right) $$

a critical assembly concentration of coat proteins, while for *c* ≤ *c*_∗_ we have *η* = 0.

Before we are in a position of investigating how the molecular weight of the polyanion impacts upon the thermodynamic stability of virus-like particles, we need to discuss how Δ*G* depends on *p* and *q*. It is important to realise that *p* sets itself, that is, it obtains an equilibrium value with an optimal value of *Q*. It seem reasonable, at this point, to assume that Δ*G* depends only on the solution conditions and the preferred curvature of the coat protein that we associate with the protein aggregation number *q*. If so, we can write *β*Δ*G* ≡ *qg* with *g* < 0 the free energy gain per coat protein in the final product, measured in units of thermal energy. Note that typical values of *g* should be in the range from − 10 to − 20 times the thermal energy [4, 14], because critical assembly concentrations are typically in the micromole per liter range [11, 20]. This allows us to write for the critical concentration

5 $$ c_{*} = \left( \frac{p}{q} \right)^{-\frac{p}{p+q}}\exp \left( \frac{q\,g}{p+q} \right).  $$

It makes sense to first consider the limit *p*/*q* ≪ 1, which corresponds to the case of actual viruses where one or a few RNAs are encapsulated in a virus particle. So, we put *p* = *O*(1) and consider the fact that many copies of the coat proteins come together to produce the capsid, *q* ≫ 1. Thus we have

6 $$ c_{*} \sim \exp \left( g\right) \left[ 1 - \frac{p}{q} \left( \ln \frac{p}{q} + g\right) + {\ldots} \right]. $$

It is immediately clear that the critical assembly concentration increases with increasing the number of encapsulated polymers *p* and thus with decreasing molecular weight of the polyanions. The effect is not very strong though, as we keep the overall mass fraction *c*_pa_/*c*_cp_ constant.

**Fig. 1:**
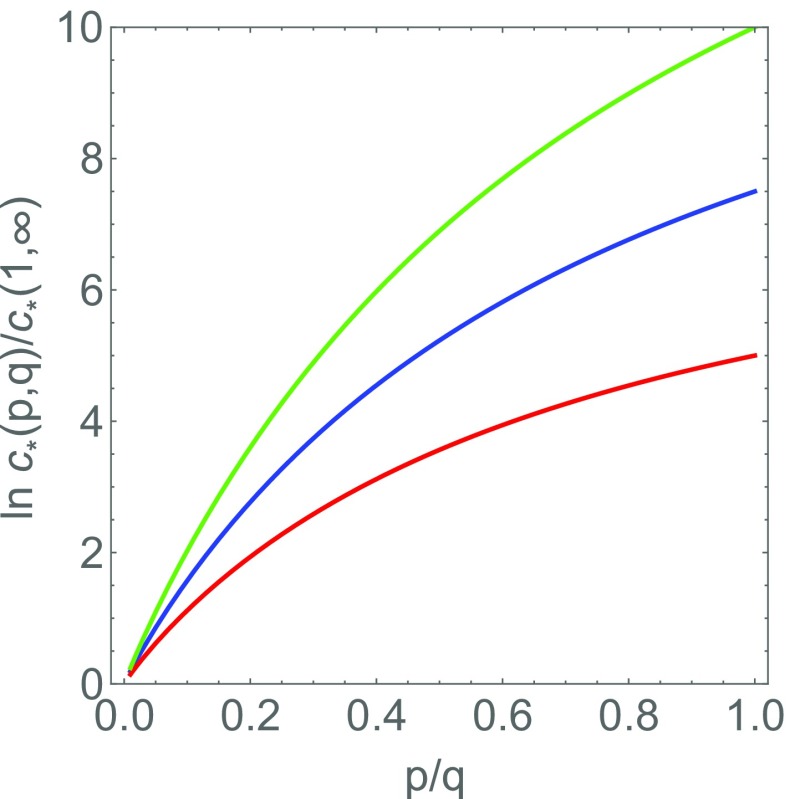
Critical protein concentrations *c*_∗_(*p*,*q*) for the formation of viruses and virus-like particles consisting of *p* encapsidated polyanions in one single shell with *q* coat proteins as a function of the ratio *p*/*q* according to the law of mass action. The stoichiometry of the solution is equal to that of the assembly. It is assumed that the overall binding free energy is equal for all *p*/*q*. The curves correspond to the binding free energies of − 20, − 15 and − 10 times the thermal energy, from top to bottom. The results are scaled to the value for the limiting case *p* = 1 and $q\rightarrow \infty $

Let us now consider the extreme case that the number of charges on one single polyanion is relatively small, of the order of the number of charges on the RNA-binding domains of the coat proteins, mimicking several recent experiments [46, 47]. For simplicity we again set *Q* = 1. Using (1) and the fact that *q* ≫ 1, we find for the cases *q*/*p* = 1,2,3,4, *c*_∗_ is equal to exp (*g*/2), 2^1/3^ exp (2*g*/3), 3^1/4^ exp (3*g*/4) and 4^1/5^ exp (4*g*/5), respectively. In this regime, the effect of molecular weight is strong. Indeed, if, e.g., exp (*g*) = 10^− 8^, then the corresponding values of *c*_∗_ would be 10^− 4^, 5.8 × 10^− 6^ , 1.3 × 10^− 6^, 5.7 × 10^− 7^ for *q*/*p* = 1,2,3,4, respectively. Actually, for *q*/*p* > 5, the critical concentration very swiftly converges to the limiting value obtained for *q*/*p* →*∞*. See Fig. 1.

Our predictions are supported by the results of Maassen et al., who find that the encapsulation efficiency of relatively short ssDNA molecules by CCMV coat proteins increases with molecular weight of the polyanion [46]. They also identify a minimum length (14 nucleotides) for co-assembly to be possible but do point out that this minimum length may be concentration dependent. In our theory, there is no minimum length, but for polyanions with a charge approaching that of the RNA binding domains, which for CCMV equals 10 charges, the critical concentration increases drastically. In the experiments of Rayaprolu et al. [47] titration curves of alphavirus coat proteins were measured as a function of the concentration of salt, not as a function of the concentration of protein. The amount of salt needed to suppress assembly increases with the length of the relatively shorts oligonucleotides, which seems to follow the predicted trend that longer oligonucleotides should produce more stable assemblies.

The findings of Cadena-Nava et al. [36] who varied the length of ssRNAs from 140 to 12,000 nucleotides, are more difficult to compare with our predictions. All the molecular weights could be packaged by CCMV coat proteins provided the mass ratio of CP to RNA was large enough, that is, corresponding to net charge neutrality. RNAs larger than 3,000 nucleotides tended to be packaged in multiplets rather than single capsids. These are outside the scope of our analysis. Unfortunately, no encapsulation efficiencies were reported for any of RNA lengths in this work. On the other hand, for the smallest RNA of 140 nt we estimate *q*/*p* ≃ 4, if we account for the fact that the coat proteins are dimers. From our theory we deduce the equivalent critical concentration of the smallest RNA in these experiments must be at most a factor of 2 larger than that of the 3000 nt RNA of which a single one is packaged in a capsid. This suggests that in these experiments the efficiencies should not have been all that different, provided the overall concentration of protein is significantly above the critical value of the smallest RNA.

Finally, the efficiencies reported on by Comas-Garcia et al. for encapsulation of long RNAs in the range from 2500 to 3600 nts, all forming *T* = 3 shells, are non-monotonic and maximal around 3200 nts [20]. For these, we expect a single polynucleotide to be encapsulated, so *p* = 1, in which case the encapsulation efficiencies must be dictated by the free energy Δ*G*. Δ*G* has been predicted to be a non-monotonic function of *N*_pa_ and exhibit a clear minimum for the optimal length [4]. This optimal length is set by a balance struck between the configurational entropy loss of a large polyanionic chain confined in a cavity and the free energy gain associated with the Coulomb interactions between the polyanionic chain and the polycationic ARM region of the shell. Clearly, this situation is also outside of the scope of our analysis.

In the next section we argue, basing ourselves on an application of a variant of the Voorn-Overbeek theory for the complexation of oppositely charged polymers [43, 44], that for *short* polyanions the optimal encapsulated amount expressed in the degree of overcharging *Q* may depend in a complicated way on both their molecular weight and the stoichiometry of the solution.

## Voorn-Overbeek theory of polyanion-ARM complexation

As far as we are aware, none of the density functional theoretic investigations of the encapsulation of the optimal length of polyanions published to date allow this length to be distributed over more than a single polyanion. Presumably, if the polyanions remain sufficiently long, we expect end effects to remain small and the optimal encapsulated length only to be weakly affected by encapsulating more than a single copy of a polyanion smaller than this optimal length [58]. This is consistent with the observation that CCMV packages the 3200 nucleotide RNA 1 and the 2800 nucleotide RNA 2 in separate particles, and co-packages RNAs 3 and 4 amounting to a total of 3000 nucleotides, in a third particle [53]. So, the total amount of length is approximately conserved over the three types of particle. Interestingly, to get equal numbers of RNA 3 and 4 in a particles in an *in vitro* co-assembly experiment, this requires very different stoichiometries of the two RNAs. The reason is that RNA 3 is almost three times the length of RNA 4, and as a consequence twice as much RNA 4 needs to be present in the solution to achieve equal stoichiometry in the virus particle. [59] As we shall see, this, in a way, could be caused again by mass action, this time influencing the binding free energy.

Now, we focus on how electrostatics and mass action determine how much polyanionic material is absorbed into the positively charged ARM regions in a capsid, that, in a thought experiment, we imagine to be pre-formed and to represent a macroscopic phase. This allows us to apply relatively straightforwardly Voorn-Overbeek theory, describing the formation of so-called complex coacervates in solutions containing oppositely charged polymers, and predict the degree of overcharging *Q* that we now do no longer consider to be some fixed number [43, 44]. Despite its known shortcomings it is generally accepted to capture the basic physics of the problem at hand [60]. The only adaptation that we need to make is to fix the concentration of the ARMs in the brush region, as our aim is to investigate how many polyanions are absorbed in polycation brush given some background concentration of polyanions of prescribed molecular weight. We shall completely ignore the chain statistics in our analysis, which should be reasonable provided the polyanions are not extremely much longer than the ARMs.

We assume that each polyanion carries *N*_pa_ negative charges, and fix their mole fraction at *ρ*_pa_ in free solution, equivalent to a volume fraction $\phi _{\text {pa}}^{\mathrm {s}}$. In our model, the background solution acts as a reservoir for the polyanions. The *N*_pc_ positive charges on the ARMs give rise to an equivalent volume fraction polycations $\phi _{\text {pc}}^{\text {arm}}$ in the polymer brush lining the inner surface of a complete capsid [5]. The positive charges attract a concentration $\phi _{\text {pa}}^{\text {arm}}$ in the ARM region of the virus particles. There are also mobile, small ionic species present in the solution and in the ARM region. These consist of the counter ions associated with the ARMs and the polyanions, as well as the ions from dissociated added salt. Let the volume fraction of mobile cations be $\phi _{+}^{\text {arm}}$ in the ARM region and $\phi _{+}^{\mathrm {s}}$ that in free solution, and likewise $\phi _{-}^{\text {arm}}$ and $\phi _{-}^{\mathrm {s}}$ for the anions. If we for simplicity presume the monomers of the ARMs and the polyanions to have the same volumes as the mobile ions, then charge neutrality in the ARM regions and in the solution demand that

7 $$ \phi_{+}^{\mathrm{s}}=\phi_{-}^{\mathrm{s}}+\phi_{\text{pa}}^{\mathrm{s}}, $$

and

8 $$ \phi_{+}^{\text{arm}}=\phi_{-}^{\text{arm}}+\phi_{\text{pa}}^{\text{arm}}-\phi_{\text{pc}}^{\text{arm}}. $$

The sources of the positively charged ions are the counter ions of the added salt and those of the polyanions. The concentrations of small anions and polyanions we consider to be independent variables in the reservoir. Hence, we fix the concentrations of the small cations by insisting on charge neutrality both in the reservoir and in the ARM region. The only positively charged species in the solution are the mobile ions, whilst the negatively charged species consist of mobile anions and polyanions. In the ARM region we have mobile cations and anions, as well as polycations and polyanions.

The mobile ions as well as the polyanions distribute themselves between the reservoir and the ARM regions of the virus-like particles in order to achieve a state of chemical equilibrium. This presumes equal chemical potentials. In the spirit of Voorn-Overbeek theory [43, 44] we calculate these, combining the Flory-Huggins theory of polymer solutions and Debye-Hückel theory of ionic solutions [57]. Presuming the reservoir to be a dilute solution and any electrostatic interactions between the constituents to be weak, we treat it as an ideal solution. This implies that the chemical potential *μ*_−_ of the negatively charged ions obeys

9 $$ \mu_{-}^{\mathrm{s}} = \ln \phi_{-}^{\mathrm{s}}, $$

in units of thermal energy, while the chemical potential of the polyanion segments is in that case given by

10 $$ \mu_{\text{pa}}^{\mathrm{s}} = \frac{1}{N_{\text{pa}}}\ln \phi_{\text{pa}}^{\mathrm{s}}, $$

where we equate the number of polymer segments with the number of charges on the backbone of the polyanion. Here, and below, we neglect the reference chemical potential as is customary at the level of Flory-Huggins theory [43, 44].

The ARM region is most definitely not dilute [32]. Indeed, taking as an example CCMV, the coat proteins of which carry a 26 residue largely disordered ARM with an estimated contour length of 10 nm and Kuhn length of 1-2 nm, suggesting an ideal solution radius of gyration of about 4 nm. With an inner capsid radius of 10 nm and 180 coat proteins we have a surface coverage of about 7 nm^2^ per ARM, implying they must be in the overlapping brush state [57]. In the virus, the complex extends approximately 3 or 4 nm into the cavity of the capsid [59]. This suggests an ARM volume fraction of the order of, say, 0.1 to 0.4.

To account for the densely packed environment in the complex brush region, we define the overall volume fraction of material $\phi _{\text {tot}}^{\text {arm}} = \phi _{+}^{\text {arm}}+\phi _{-}^{\text {arm}}+\phi _{\text {pc}}^{\text {arm}}+\phi _{\text {pa}}^{\text {arm}}$. For the mobile anions we then obtain for the chemical potential the expression [43, 44]

11 $$ \mu_{-}^{\text{arm}} = \ln \phi_{-}^{\text{arm}}-\ln \left( 1- \phi_{\text{tot}}^{\text{arm}} \right) - a \sqrt{\phi_{\text{tot}}^{\text{arm}}}, $$

while for the polyanions in the ARM region we have for the chemical potential per segment

12 $$ \mu_{\text{pa}}^{\text{arm}} =\frac{1}{N_{\text{pa}}} \ln \phi_{\text{pa}}^{\text{arm}} -\ln \left( 1- \phi_{\text{tot}}^{\text{arm}} \right)- a \sqrt{\phi_{\text{tot}}^{\text{arm}}}. $$

In both expressions, the second term on the right-hand side stems from excluded-volume interactions and the third results from the electrostatic interactions with the various charged species at the level of the Debye-Hückel approximation. The net attractive interaction is caused by the correlations between positively and negatively charged species in the solution, but ignore any chain connectivity [60]. The constant $a\equiv \sqrt {\pi } \lambda _{B}^{3/2}/ l^{3/2} \approx 1$ is a function of the ratio of the Bjerrum length *λ*_*B*_ and the Kuhn length or size of a statistical polymer segment, which we have tacitly set equal to the size of the solvent molecules and the mobile ions. *l* must be of the order of 1 − 3 nm. The Bjerrum length is the distance over which the Coulomb energy of two elementary charges in the liquid medium is equal to the thermal energy. In water at room temperature, *λ*_*B*_ = 0.7 nm [57]. We assumed for simplicity that the polycations and polyanions carry a single elementary charge per polymer segment.

Chemical equilibrium of the mobile ions in the reservoir and those in the ARM region demands that $\mu _{-}^{\mathrm {s}}=\mu _{-}^{\text {arm}}$, and similarly equal chemical potentials for the polyanions in the reservoir and ARM region imposes the equality $\mu _{\text {pa}}^{\mathrm {s}}=\mu _{\text {pa}}^{\text {arm}}$. This immediately gives for the adsorption isotherms for the negatively charged species the following expressions

13 $$ \frac{\phi_{\text{pa}}^{\text{arm}}}{\phi_{\text{pa}}^{\mathrm{s}}}= \left( 1- \phi_{\text{tot}}^{\text{arm}} \right)^{N_{\text{pa}}} \exp \left[ a N_{\text{pa}} \sqrt{\phi_{\text{tot}}^{\text{arm}}}\right], $$

and

14 $$ \frac{\phi_{\textrm{-}}^{\text{arm}}}{\phi_{\textrm{-}}^{\mathrm{s}}}= \left( 1- \phi_{\text{tot}}^{\text{arm}} \right) \exp \left[ a \sqrt{\phi_{\text{tot}}^{\text{arm}}} \right]. $$

In other words,

15 $$ \left( \frac{\phi_{\text{pa}}^{\text{arm}}}{\phi_{\text{pa}}^{\mathrm{s}}}\right) =\left( \frac{\phi_{-}^{\text{arm}}}{\phi_{-}^{\mathrm{s}}}\right)^{N_{\text{pa}}}. $$

Because we expect $\phi _{-}^{\text {arm}}/ \phi _{-}^{\mathrm {s}} \geq 1$, which is true so long as excluded volume interactions do not suppress accumulation of ions in the brush region, we find that the larger *N*_pa_ is, the larger the accumulation of the negatively charged species in the ARM region becomes, and hence the larger the free energy gain. Within Debye-Hückel theory, the free energy gain of inserting a single polyanion in the ARM region scales as $N_{\text {pa}} a (\phi _{\text {tot}}^{\text {arm}})^{3/2}$: the number of positive charges per polyanion times the free energy of inserting a single charge [43, 44, 60]. As our Voorn-Overbeek model of polymer ad- or, rather, absorption, ignores partially embedded chains, i.e., loops and ends protruding into the cavity of the capsid, we expect the model to become more accurate the shorter the chains are. Because of this, we cannot expect our theory to hold if *N*_pa_ ≫ *N*_pc_, that is, for polyanions large enough to compensate a large fraction of the total charge in the ARM region of the capsid. We note that an analysis based on a Donnan equilibrium would produce a very similar outcome (results not shown) [61, 62].

**Fig. 2:**
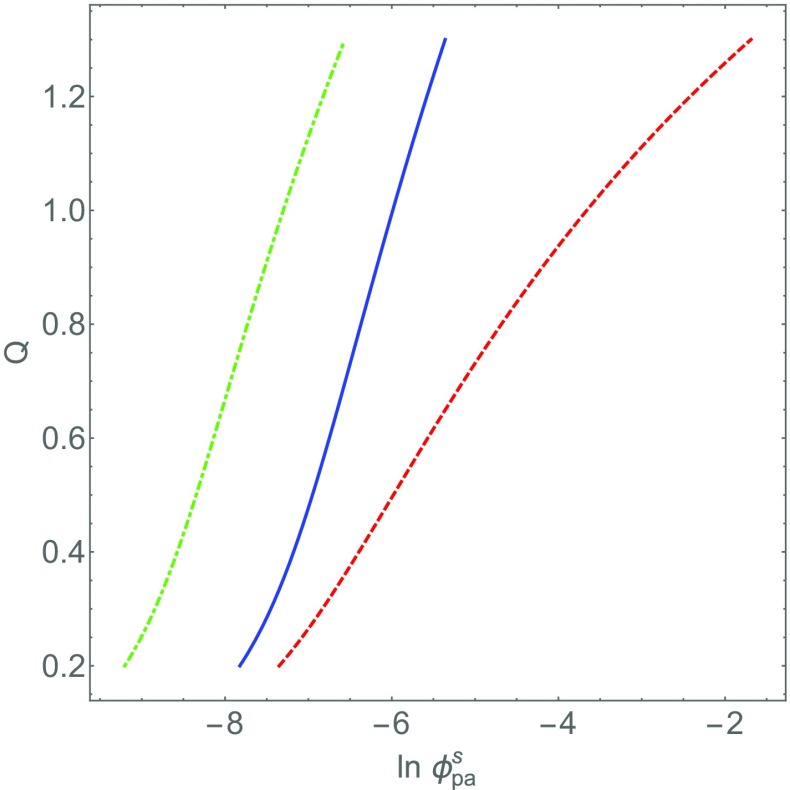
Degree of overcharging *Q* taking place in the polycation brush as a function of the volume fraction of polyanion $\phi _{\text {pa}}^{\mathrm {s}}$ in the solution within the Voorn-Overbeek model described in the main text. The solid (blue) curve corresponds to a polycation volume fraction $\phi _{\text {pc}}^{\text {arm}} = 0.2$ in the RNA binding region of the capsid and a polyanion charge of $N_{\text {pa}}^{-}= 10$. The dashed (red) curve is that of the case $\phi _{\text {pc}}^{\text {arm}} = 0.3$ and $N_{\text {pa}}^{-}= 10$, while for the dash-dotted (green) curve $\phi _{\text {pc}}^{\text {arm}} = 0.2$ $N_{\text {pa}}^{-}= 13$. We set the Bjerrum length equal to half the Kuhn length of the chains

A number of important conclusions can now be drawn. First, the theory suggests that longer polyanions are preferentially absorbed in the ARM region and hence should be preferentially encapsulated. This strengthens the effects of mass action discussed in the previous section. Second, the degree of overcharging $Q = \phi _{\text {pa}}^{\text {arm}}/\phi _{\text {pc}}^{\text {arm}}$ that we obtain from (13) can be written in terms of the following self-consistent equation

16 $$ Q = \left( \frac{\phi_{\text{pa}}^{\mathrm{s}}}{ \phi_{\text{pc}}^{\text{arm}}} \right) \exp \left[ N_{\text{pa}} \left( a \sqrt{\phi_{\text{tot}}^{\text{arm}}} + \ln \left( 1- \phi_{\text{tot}}^{\text{arm}} \right)\right) \right], $$

where the overall concentration of charges in the ARM region, $\phi _{\text {tot}}^{\text {arm}} = \phi _{+}^{\text {arm}}+\phi _{-}^{\text {arm}}+\phi _{\text {pc}}^{\text {arm}}+\phi _{\text {pa}}^{\text {arm}}$, to a good approximation obeys

17 $$ \phi_{\text{tot}}^{\text{arm}}=\phi_{+}^{\text{arm}} + \phi_{-}^{\text{arm}} + \phi_{\text{pc}}^{\text{arm}} \left( 1 + Q\right) = \phi_{\text{pc}}^{\text{arm}} \left( 1 + Q\right), $$

because we expect $\phi _{+}^{\text {arm}} + \phi _{-}^{\text {arm}} \ll \phi _{\text {pc}}^{\text {arm}}\left (1 + Q\right )$. In Fig. 2 we have plotted *Q* as a function of *ϕ*_pa_, for different values of *N*_pa_ and $\phi _{\text {pc}}^{\text {arm}}$.

As a matter of fact, we need not solve (16) to be able to draw conclusions. First, the degree of overcharging *Q* is strongly reduced by excluded volume interactions in the ARM region due to the logarithmic term in the exponent, in particular if $\phi _{\text {tot}}^{\text {arm}} \rightarrow 1$. Second, the concentration of free polyanions in the solution $\phi _{\text {pa}}^{\mathrm {s}}$ also affects the degree of overcharging: the larger $\phi _{\text {pa}}^{\mathrm {s}}$ the larger *Q*. This is a direct consequence of mass action. Third, increasing the number of charges *N*_pa_ on the polyanion strongly enhances the degree of overcharging, of course within the bounds of applicability of the model. See the discussion above. The results of Fig. 2 confirm this.

What we can conclude from the present and the previous section, is that mass action acts on different levels in the problem of the encapsulation of virus coat proteins and polyanions. On the one hand, for a given overall encapsulation free energy, it works against the encapsulation of short polyanions. This is true even if the mass stoichiometry remains fixed, that is, under assembly conditions where the ratio of the total number of positive charges on the ARMs and negative charges on the polyanions is equal. On the other hand, mass action works also against the complexation of short polyanions with the polycationic RNA binding domain of the coat protein. This means that the free energy of encapsulation is in fact not fixed, but increases with decreasing molecular weight of the polyanion. Hence, both effects conspire to favour encapsulation of longer polyanions.

## Parasitic complexation with host proteins

We have seen that mass action should very much act against the encapsulation non-viral RNAs in infected cells, if only because viral RNAs tend to be very much larger than cellular RNAs [41]. The question arises whether nonspecific binding of cellular proteins to coat proteins could significantly affect the efficiency of virus assembly, if it is not compartmentalised, e.g., in a “virus factory”, and screened from contact with the cytosol [42]. The cytosol is, as already advertised, awash with proteins. The polycationic domains of coat proteins could bind to other proteins even if their net charge is positive, and form, e.g., dimers, trimers, and so on, rather than complete shells. (Of course, cellullar RNAs could also bind to the ARMs and not to form a shell.) The only requirement, at least *in vitro*, is to have a sufficiently large number of negative charges on the surface of that protein and low enough concentration of salt [63].

The question that we wish to address is to what extent the assembly of viruses or virus-like particles could be suppressed if the coat proteins potentially form dimers with other proteins present in the solution. We presume the coat protein to encapsulate only a single viral RNA molecule. For definiteness, we also presume, as before, that the overall stoichiometry of the RNA and coat protein in the solution is that of the virus: this implies that their concentrations obey *c*_pa_/*c*_cp_ = 1/*q* with *q* ≫ 1 as before the number of proteins in a complete capsid. Let there be *n* ≥ 1 other kinds of (host) protein in the solution that may bind to coat proteins and form dimers. Their overall concentration we denote *c*_*i*_ = *ρ*_*i*_ + *ρ*_*i*,*c*_, which is the sum of the concentration *ρ*_*i*_ of host proteins in free solution and the concentration *ρ*_*i*,*c*_ of that bound to coat protein. The dimensionless binding free energy we denote *g*_*i*_ ≤ 0, as before in units of thermal energy. We expect *g*_*i*_ ≈−*O*(1).

According to the law of mass action, the fraction of coat proteins $f\equiv {\sum }_{i = 1}^{n} \rho _{i,c} / c_{\text {cp}}$ bound to the other proteins in dimers obeys

18 $$ f = \left( 1-f-\eta\right)\sum\limits_{i = 1}^{n} \left( c_{i} - \rho_{i,c}\right) \exp \left( -g_{i}\right), $$

which can be obtained by if the solution to be dilute, equating chemical potentials of the coat proteins in dimers and in free solution, and making use of the conservation of mass. [57] Here, we have specifically accounted for a fraction *η* of the coat proteins present in capsids, which obeys a mass action equation very similar to that of (2),

19 $$ \eta = c_{\text{cp}}^{q} \left( 1- f - \eta \right)^{q} \left( 1-\eta\right)\exp \left( - q g \right), $$

where *g* as before indicates the dimensionless binding free energy of a virus-like particle per coat protein, i.e., we set *β*Δ*G* = *qg*. The difference between (2) and (19) is that in the latter one we have further accounted for the coat protein bound in dimeric complexes with cellular proteins.

Equations (18) and (19) need to be solved self-consistently. This can be done analytically in the limit *q* ≫ 1 provided we make the preaveraging approximation ${\sum }_{i=n}^{n}$ (*c*_*i*_ − *ρ*_*i*,*c*_) exp (−*g*_*i*_)≈$ \exp \left (-h\right ) \times {\sum }_{i = 1}^{n} \left (c_{i} - \rho _{i,c}\right )$ with $\exp \left (-h\right ) \equiv n^{-1} {\sum }_{i = 1}^{n} \exp \left (-g_{i} \right )$ the average of the Boltzmann weights of the binding free energies of the various species of protein engaging in parasitic binding. The law of mass action describing the parasitic binding, (18), then simplifies to

20 $$ f = \left( 1-f-\eta\right)\left( c_{\mathrm{p}} - c_{\text{cp}} f \right) \exp \left( -h\right), $$

with $c_{\mathrm {p}} \equiv {\sum }_{i = 1}^{n} c_{i}$ the overall concentration (mole fraction) of non-viral (host) proteins present in the solution.

Taking the 1/*q* power of the left- and right-hand side of (19), making use of the limit *q* ≫ 1 and inserting the resulting equation in (20), the expression for the fraction of coat proteins in dimers can be simplified to

21 $$ f = \frac{ 1}{ 1 + \exp \left( h - g \right)} \left( \frac{c_{\mathrm{p}}}{c_{\text{cp}}}\right) , $$

under conditions where |ln (*η*/(1 − *η*))|≪ *q*, so for conditions where *η* is not vanishingly small nor very close to unity. Of course, if *f* → 1 these conditions no longer hold, so we have to insist that *f* < 1. For the fraction of coat proteins in virus particles we retrieve (3), that is, *η* = 1 − *c*_∗_/*c*_cp_ for *c*_cp_ ≥ *c*_∗_ and *η* = 0 if *c*_cp_ ≤ *c*_∗_, with an effective critical concentration *c*_∗_ = *c*_∗_(*c*_p_) that obeys

22 $$ c_{*} = \exp \left( g\right) + \frac{ c_{\mathrm{p}}}{1 + \exp \left( h -g\right)}. $$

Apparently, the critical concentration now depends on the concentration of host protein, *c*_p_. We recall that we presume the solution to be dilute, so *c*_cp_ + *c*_p_ ≪ 1, and that the “bare” critical concentration, in the absence of parasitic binding, or, equivalently, if *c*_p_ → 0, is given by (6). In that case, the critical concentration reduces to exp (*g*) to leading order in 1/*q* for *p* = 1 and *q* ≫ 1.

**Fig. 3:**
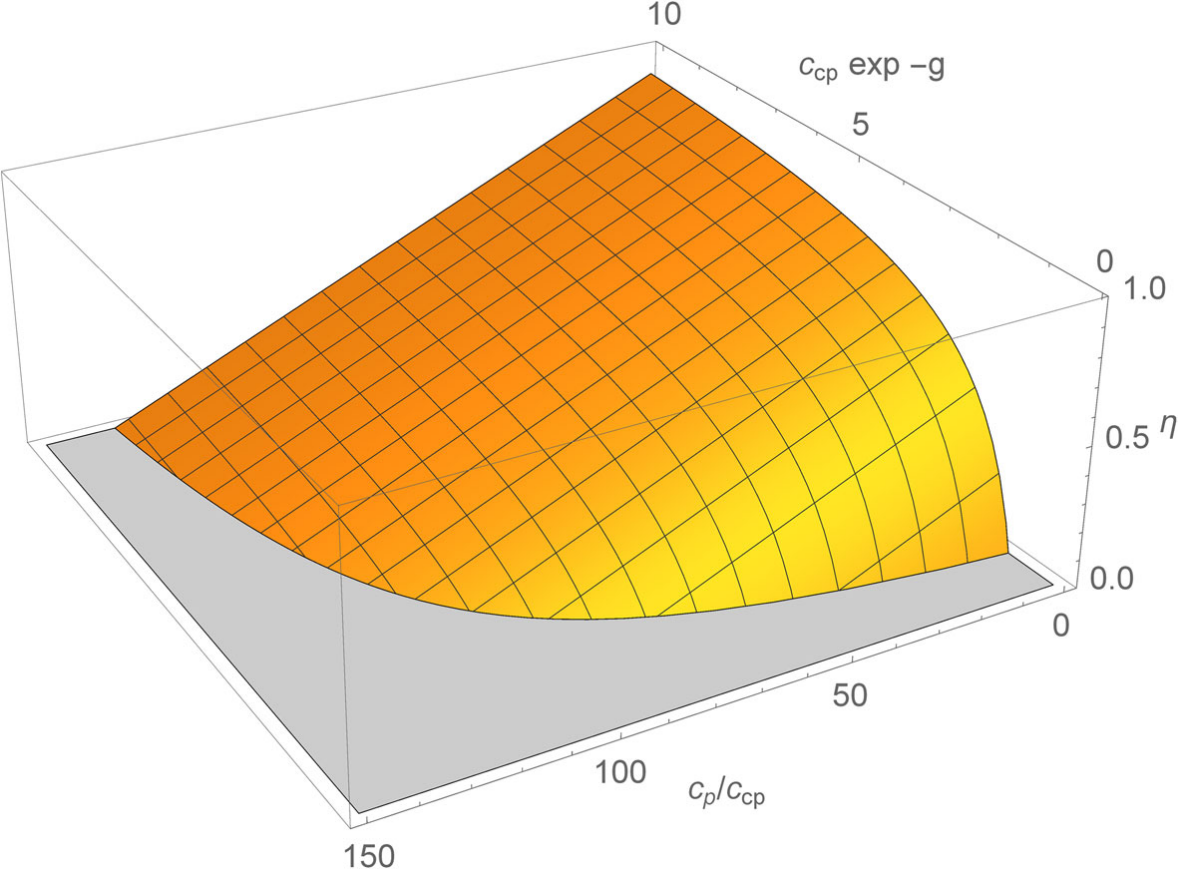
Fraction of coat proteins in virus particles *η* as a function of the scaled concentration $c_{\text {cp}}\exp (-g)$ and the ratio of concentrations of host proteins and coat proteins *c*_p_/*c*_cp_ according to the law of mass action. Here, *g* denotes the dimensionless binding free energy of a single coat protein in a virus particle that has encapsulated a single polyanion, the concentration of which is set to reproduce the stoichiometry of proteins and polyanion in a virus particle. The host and coat proteins are able to dimerise, and liberate a binding free energy *h*. For definiteness, we set *h* = *g* + 5 and took the formal limit $q\rightarrow \infty $ for the aggregation number *q* of the virus shell

From these expressions, we are able to conclude that the fraction of coat proteins in dimers with host proteins increases with increasing the ratio of host protein to coat protein. This, again, is to be expected because it is a consequence of the law of mass action. The effective critical concentration increases with increasing concentration of coat proteins, even if *h* is much less negative than *g*. The presence of host proteins shifts the amount of coat proteins required to form thermodynamically stable virus particles, as is illustrated in Fig. 3. The reason for this is the difference in the level of co-opperativity between dimerization and capsid shell assembly. Indeed, capsid assembly is highly co-operative and is characterized by a sharp polymerization transition, where below a well-defined critical concentration, *c*_∗_, assembly does not take place. Simple dimerization is a very much more gradual function of the concentration of both components, so it already happens at concentrations well below the value for which half the material presents itself in dimers. From (20) we find that for *η* = 0, in the absence of capsid formation, *f* = 1/2 if the host protein concentration *c*_p_ is equal to exp (*h*) + *c*_cp_/2. If virus particles do form, and *η* > 0, (21) tells us that *f* = 1/2 for very much larger host protein concentrations *c*_p_ equal to (1 + exp(*h* − *g*))*c*_cp_/2, noting that exp(*h* − *g*) ≫ 1.

We furthermore conclude from these calculations that if *c*_p_ > (*c*_cp_ − exp(*g*))(1 + exp(*h* − *g*)) ∼ *c*_cp_(1 + exp(*h* − *g*)) for *c*_cp_ ≫ exp(*g*) no virus particles are able to form, even if *c*_cp_ ≫ exp(*g*) and capsids would form in the absence of host proteins. Under these conditions *f* → 1, so all coat proteins are bound to host proteins and (21) strictly speaking no longer holds. To confirm these findings, we can take the formal limit *η* → 0 and insert *f* = 1 − *δ* where *δ* ≪ 1 into (19) and (20), and evaluate the leading order behaviour of *δ*. We find the following asymptotic relations for *η* ∼(*δc*_cp_exp(−*g*))^*q*^ ≪ 1 and *δ*^− 1^ ∼ 1 + (*c*_p_ − *c*_cp_)exp(−*h*) ≫ 1. Since *q* ≫ 1, we only need to insist that *η* vanishes for *δc*_cp_exp(−*g*) < 1. This is the case provided *c*_p_ > *c*_cp_(1 + exp(*h* − *g*)) − exp(*h*), which agrees with the previous one since *h* is negative and at least of the order of unity in magnitude, or otherwise dimers would not form.

It is informative to turn the mathematics into actual numbers. Plausibly, − *g* is of the order of ten to twenty times the thermal energy *k*_*B*_*T* and − *h* a few times the thermal energy. From this, we expect *c*_p_/*c*_cp_ > 10^4^ for complete suppression of virus assembly to happen. In *in vitro* experiments, *c*_cp_ is typically of the order of 10^− 6^, suggesting that *c*_p_ should be of the order 10^− 2^ or, equivalent, to 0.6 M for parasitic binding to completely take over. For the average cell protein of 40 kDa this corresponds to about 24 kg per liter, which, of course, is absurd. This can only mean that we should expect parasitic binding of coat proteins to host cell proteins never to substantially impact virus assembly, even if the host proteins overwhelm the coat proteins in terms of abundance in the environment where virus assembly takes place. With hindsight this is not an unexpected conclusion, considering that the concentrations of the constituents enter the law of mass action, describing the expectation value of the concentration of an assembly, algebraically whilst binding free energies enter exponentially. This means that differences in binding free energy of more than a few times the thermal energy are very difficult to overcome in competing assembly pathways, as they require order of magnitude differences in concentration.

## Discussion and conclusions

The *in vitro* self-assembly of viruses and of virus-like particles is driven by net attractive interactions between coat proteins and RNA molecules (or the polyanions replacing the RNA molecules) and opposed by the loss of mixing of translational entropy of the constituents. The law of mass action that is the result of this balancing act stipulates that the larger the concentration of both constituents, the larger the relative amount of complete particles will be. Our model calculations have made clear that because complete virus particles require two constituents to come together, mass action enters the physics of the problem not at one level but at least at two levels. First, it enters directly in the statistics of the competition between assembled and disassembled states for a given binding free energy per particle. Second, it enters via the binding free energy itself that is a function of the concentrations of the molecules involved.

Within our simplified model description, both effects act in favour of the encapsulation of larger molecular weights of polyanion at least up to the optimal molecular weight for a single encapsulated polyanion. This is consistent with recent experimental findings [46, 47]. It could explain why in *in vivo* assembly, cellular RNAs that present in large amounts in the cytosol do not outcompete viral RNAs that typically are very much longer [41]. Mass action could also explain why parasitic binding of coat proteins to host proteins does also not significantly impact the efficiency of virus assembly. Here, the relatively large binding energy of coat proteins associated with forming a closed shell containing many tens to hundreds of proteins requires unrealistically large quantities of host protein to counter this.

Clearly the assembly statistics and the binding free energy gain of co-assembly need to be accounted for self-consistently, as they are two faces of the same mass action phenomenon. We have not attempted to do this, because it requires a very much more sophisticated absorption theory than the Voorn-Overbeek model that we have employed. The more sophisticated theory has to deal with the intricacies of the complexation of polyanions with a reasonably dense brush of polycationic ARMS [5]. In principle, one would have to go beyond the ground-state theory and deal with finite-size effects under conditions of confinement in a spherical cavity. Even for the relatively simple case of adsorption of a polymer onto a flat surface, this is a formidable task [58]. Incidentally, the confinement will work against encapsulation of very long chains, that is, much longer than the optimal length [4]. This has been confirmed experimentally: Comas-Garcia et al. found a non-monotonic dependence of the encapsulation efficiency on the length of single-stranded RNA too large to encapsulate more than a single copy [20].

Finally, if virus assembly is kinetically rather than thermodynamically controlled then the efficiency of co-assembly is no longer dictated by the law of mass action [38]. Still, kinetics should also work against the encapsulation of shorter polyanions in mixtures. Indeed, Cornelissen et al. find that encapsulation of oligonucleotides by virus coat proteins of a simple plant virus is faster and more efficient for longer oligonucleotides [46]. That it should be faster (and more efficient) is perhaps to be expected also in the light of our findings. First, as larger polyanions require fewer molecules to come together to form a virus-like particle, and this should therefore be faster through a lower order type of reaction. Second, according to our calculations, the thermodynamic driving force for assembly is larger for longer polyanions, and this should also speed up assembly and increase its efficiency [64]. Again, these conclusions apply to relative short polyanions, much shorter than the optimal overall length of polyanion required to optimise the co-assembly.
